# Fungal community remediate quartz tailings soil under plant combined with urban sludge treatments

**DOI:** 10.3389/fmicb.2023.1160960

**Published:** 2023-04-20

**Authors:** Fabao Dong, Yujia Zhu, Xunmei Zhu, Chengzhi Zhang, Yingying Tao, Taotao Shao, Yue Wang, Xia Luo

**Affiliations:** ^1^School of Biological Science and Food Engineering, Chuzhou University, Chuzhou, Anhui, China; ^2^Center of Excellence in Fungal Research, Mae Fah Luang University, Chiang Rai, Thailand; ^3^School of Science, Mae Fah Luang University, Chiang Rai, Thailand; ^4^College of Food and Pharmaceutical Engineering, Guizhou Institute of Technology, Guiyang, Guizhou, China

**Keywords:** fungal diversity, fungal community structure, phytoremediation, quartz tailings, phosphorus and nitrogen, urban sludge

## Abstract

**Introduction:**

Tailings can cause extensive damage to soil structure and microbial community. Phytoremediation is an effective strategy for remedied tailings soil due to its environmentally friendly and low-cost advantage. Fungi play a crucial role in nutrient cycling, stress resistance, stabilizing soil structure, and promoting plant growth. However, the fungal community variation in phytoremediation remains largely unexplored.

**Methods:**

We analyzed soil fungal community based on high-throughput sequencing during three plant species combined with urban sludge to remediate quartz tailings soil.

**Results:**

The results indicated that the fungal diversity was significantly increased with plant diversity, and the highest fungal diversity was in the three plant species combination treatments. Moreover, the fungal diversity was significantly decreased with the addition of urban sludge compared with plant treatments, while the abundance of potential beneficial fungi such as *Cutaneotrichosporon*, *Apiotrichum*, and *Alternaria* were increased. Notably, the fungal community composition in different plant species combination treatments were significant difference at the genus level. The addition of urban sludge increased pH, available phosphorus (AP), and available nitrogen (AN) content that were the main drivers for fungal community composition. Furthermore, the fungal networks of the plant treatments had more nodes and edges, higher connectedness, and lower modularity than plant combined with urban sludge treatments.

**Conclusion:**

Our results showed that three plant species combined with urban sludge treatments improved fungal community and soil properties. Our results provide insights for quartz tailings soil remediation using plant-fungi- urban sludge.

## 1. Introduction

The exploitation of mineral resources that play an essential role in the development of humans was shown exponential growth as the economy development (Carvalho, 2017). However, the vast amount of waste liquids and rocks from the mining process are transported into soil and then destroy the ecological environment (Kossoff et al., 2014; Adiansyah et al., 2015). It is difficult for plants to grow on tailings soil due to their physicochemical characteristics, such as lower or higher pH, higher heavy metal pollution, lower water retention capacity, deficiencies in soil organic matter, and lack of plant growth nutrients (Sheoran et al., 2008; Wang et al., 2017; da Silva et al., 2022). Notably, the changes in soil physicochemical properties can affect the soil microbial community structure (Jia et al., 2019). The common restoration methods for tailings soil are biological, physical, and chemical remediation (Festin et al., 2018). Phytoremediation including plants and related microorganisms that have the advantages of low-cost, sustainable, and environmentally friendly technology, are ideally used to remediate tailings soil compared with physical and chemical remediation (Dickinson, 2017; Sharma, 2020).

More than 400 kinds of plant species in the world can metabolize pollutants including accumulating organic and inorganic contaminants by improving microbial degradation of contaminants in the root zone (Arthur et al., 2005; Faucon et al., 2007). Furthermore, plants can transport, accumulate or degrade soil heavy mental pollutions such as Zn, Pb, Cd, Mn, Cu, Cr, Fe, As, and Ni, and then reduce environmental impact through litter decomposition, root exudates and soil properties (Wang et al., 2015; Sanchez et al., 2018; Ali et al., 2020; Su et al., 2022). For example, some species of Legumes are often used to improve the nitrogen content of tailings soils (Young et al., 2015). Moreover, some plant species can tolerate drought of semiarid and mining soil with low water holding capacity (Santibañez et al., 2012). Therefore, suitable plant species are very important to repair tailings soil.

Fungal community are critical factors in maintaining plant biodiversity, soil health and productivity, and soil biogeochemical processes such as nitrogen and phosphorus cycling (Mangan et al., 2010; Urbanová et al., 2015). They contribute to plant disease control and growth promotion, and plant stress resistance (Ozimek and Hanaka, 2020). Some fungal groups, such as *Penicillium*, can assist plant in phosphorus and nitrogen uptake in heavy metal-contaminated soil (Ikram et al., 2018; Elfiati et al., 2021). Moreover, some species of Basidiomycetes can improve plant salt tolerance by increasing the concentration of osmotic fluid in plant cells, and enhancing plan minerals uptake and potassium ions for metal-detoxifying (Bukhori et al., 2020). Notably, arbuscular mycorrhizal fungi (AMF) can assist plant to facilitate mineral and water uptake (Debeljak et al., 2018; Begum et al., 2019). *Archaeorhizomyces* enhance stress resistance and inhibit disease by increasing the bioactive components of the plant (Zhang et al., 2020). Furthermore, the plant growth promoting fungi (PGPR) have been widely used to prevent and control heavy metal pollution due to its ability to promote plant growth and induce host resistance (Geetha et al., 2022). Moreover, fungi also play a crucial role in maintaining soil plant productivity (Frac et al., 2018). These findings suggest that fungal community play an essential role in plant defense against stressful environment and facilitate nutrient absorption. In turn, plant can significantly improve the diversity and abundance of soil microbial community by providing an ideal environment to inhabit (Zhao A. et al., 2019; Wu et al., 2022). Plant can recruit beneficial fungal community through root systems, stems, leaves, and even seeds (Santoyo, 2021). Higher microbial diversity led to higher microbial function level and then accelerate tailings soil remediation (Fuke et al., 2021). However, plant recruit which fungal community and play what roles in tailings soil remediation remain unclear.

Extreme environmental conditions strongly influence the efficiency of phytoremediation (Chandra and Kumar, 2018). In order to improve the recovery efficiency, some amendment combination with plants have been used in the rehabilitation of tailings soil (Asensio et al., 2013b; Alcantara et al., 2015). Notably, some studies have shown that urban sludge significantly neutralized soil pH in restoring Cu tailings (Asensio et al., 2013a). In addition, urban sludge can improve soil properties and influence soil bacterial community structure (Bai et al., 2019; Zuo et al., 2019). Significantly, urban sludge is often used as an amendment to phytoremediation due to its easy availability and rich in inorganic and organic nutrients. However, we still need to better understand how the plant species and urban sludge improve the properties and biological condition of tailings soil. In this study, we used three plant species (*Lolium perenne* L., *Vicia sepium* L., and *Medicago sativa* L.) combined with urban sludge to remediate quartz tailings soil. We analyzed the variation and co-occurrence of fungal community in 14 treatments. We hypothesized that (i) the treatments of plant species combination could significantly increase fungal diversity than single species. (ii) Plant could recruit beneficial fungi to resist stress in tailings soil. (iii) Plant combined with urban sludge could effectively remediate tailings soil due to the improvement of biological-chemical-physical properties. The results provide systematic information for the remediation of tailings soil and fully reveal the relationship among plant, urban sludge, and soil fungal community.

## 2. Materials and methods

### 2.1. Material preparation

Quartz tailings soil and urban sludge were collected separately from the Fengyang County mining area and Chuzhou Zhongye Huatian Water Co., Ltd. of Chuzhou City, Anhui Province, China. Impurities on the surface of quartz tailings soil were removed during sampling, and quartz tailings soil were randomly collected and brought back to the laboratory for remediation test. Three soil samples were spread in a ventilated place to dry, and then passed through 2 mm sieve to remove impurities. The sieved soil samples were used to measure soil physical and chemical properties. The urban sludge reached the discharge standard after being treated by the providing company, and the properties such as nutrients and harmful substances meet the Chinese national standards.

*Vicia sepium* L.(Y) and *M. sativa* L.(Z) belong to Legume that have the characteristic of drought and barren tolerance, salt and alkali resistance, and strong ability to enrich heavy metals (Templeton, 2018; Bao et al., 2022). *Lolium perenne* L.(H) is widely used in tailings soil due to its strong resistance and growth ability (Irhema, 2019). We used the three plant species to remediate tailings soil. The seeds of the three plant species were purchased from Century Tianyuan (Luoyang) Ecological Technology Co., Ltd. (Henan Province, China). Seeds were soaked in sterile water for 12 h at room temperature, then loaded into sterilized Petri dishes, and covered with wet gauze until germination. Seedlings that were strong and uniform in size were selected for the subsequent treatment.

### 2.2. Experimental design

**FIGURE 1:**
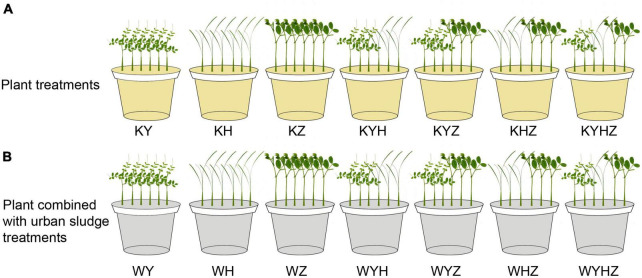
Experimental design diagram of quartz tailings soil remediation by plant and plant combined with urban sludge. Panel **(A)** represents plant treatments. Panel **(B)** represents plant combined with urban sludge treatments. Y, H, and Z represent *Vicia sepium* L., *Lolium perenne* L., and *Medicago sativa* L., respectively.

The experiment was conducted in a greenhouse using potted plants placed in polyethylene plastic pots (upper diameter: 16 cm, lower diameter: 12 cm, height: 17 cm, volume: 2 L). Approximately 1 kg of quartz tailing soil was placed in each pot. Quartz tailings soil was treated with plant or plant combined with urban sludge, respectively. The treatments of plant species were divided into one, two and three plant species combination. Therefore, seven treatments were set in the plant treatment group, including Y treatment (KY), H treatment (KH), Z treatment (KZ), Y & H treatment (KYH), Y & Z treatment (KYZ), H & Z treatment (KHZ), and Y & H & Z treatment (KYHZ), respectively, (Figure 1A). Seven plant species combined with urban sludge treatments were set in Figure 1B. Two hundred gram of urban sludge were drizzled in the quartz tailing soil in each pot. Seven treatments in plant combined with urban sludge group were Y treatment (WY), H treatment (WH), Z treatment (WZ), Y & H treatment (WYH), Y & Z treatment (WYZ), H & Z treatment (WHZ), and Y & H & Z treatment (WYHZ), respectively. Six uniform seedlings were planted per pot. Three or two seedlings of each plant species in two plants and three plants mixed treatments were planted per pot, respectively. Each treatment was replicated five times. Watering was in demand during 100 days of treatment.

### 2.3. Quartz tailings soil properties and plant biomass analysis

Soil of 14 treatments were collected and sieved through a 2 mm sieve after the experiment, and then divided into two parts for soil properties determination and DNA extraction. The content of total nitrogen (TN), available phosphorus (AP) and nitrogen (AN), iron trioxide (Fe_2_O_3_), pH, and silicon dioxide (SiO_2_) were measured according to the method of previous studies (Sun et al., 2015; Qi et al., 2017; Yu et al., 2017; Zhao S. et al., 2019). Briefly, pH was determined by pH meter (S500-F, Mettler Toledo, Germany) at a ratio of 1:2.5 (weight/volume) for soil versus distilled water. The content of TN was determined by the Kjeldahl digestion method. AP was extracted using 0.5 M NaHCO_3_ and determined by molybdenum blue method. Alkaline hydrolysis diffusion was used to determine the soil AN. The content of Fe_2_O_3_ in soil was determined by atomic absorption spectroscopy. The content of SiO_2_ was determined by UV-visible spectrophotometry. Furthermore, plants of each pot were harvested, and measured the aboveground and belowground biomass of each plant.

### 2.4. DNA extraction and sequencing

DNA of soil samples were extracted using the E. Z.N.A™ Mag-Bind Soil DNA Kit (Omega, United States). DNA quality was checked using 1% agarose gel electrophoresis and stored at −80°C for PCR amplification. The fungal ITS1 region was amplified using the primer ITS1F (5′-CTTGGTCATTTAGAGGAAGTAA-3′) and ITS2R (5′-GCTGCGTTCTTCATCGATGC-3′) on ABI GeneAmp 9,700 PCR thermocycler (ABI, CA, United States). The PCR products were recovered by cutting the gel using the AxyPrep DNA Gel Recovery Kit (Axygen Biosciences, United States), and then detection and quantification were performed with the QuantiFluor™-ST blue fluorescence quantitative system (Promega, United States). Paired-end sequencing were performed by Majorbio Bio-Pharm Technology Co. Ltd. (Shanghai, China) on the Illumina MiSeq PE300 platform (Illumina, San Diego, CA, United States). All raw reads were deposited into NCBI Sequence Read Archive (SRA) database (Accession number: PRJNA923135). The raw sequences that contained ambiguous nucleotides, short length, and low quality were discarded (Sun et al., 2021). The high-quality sequences were analyzed by the QIIME (Caporaso et al., 2010). Operational taxonomic units (OTUs) at 97% similarity were identified using the UPARSE (Edgar, 2013). All sample sequences were flattened according to the minimum sequence number. 21,160 OTUs were acquired, and flattened at the OTUs level. OTUs scale generated after flattened were used for subsequent analysis.

### 2.5. Bioinformatics and statistical analysis

Alpha diversity indicators including Chao, and Shannon index were calculated using the vegan package in *R* at the OTUs level. The difference among treatments were analyzed using Student’s *t*-test significant difference (Kembel et al., 2010). Fungal community composition at the phylum and genus level were generated using the “ggplot2” package (Villanueva and Chen, 2019). The difference of fungal community composition were evaluated using principal coordinate analysis (PCoA) based on the Bray-Curtis distance (Edgar, 2013). The relationship between fungal community and soil properties were analyzed based on Redundancy analysis (RDA). The relationship between soil properties and fungal diversity were performed using Pearson’s correlation analyses (the top 20 at the genus level) (Capblancq and Forester, 2021; Cheng et al., 2021). Significant difference of the relative abundance of fungal community among different treatments were analyzed using the linear discriminant analysis effect size (LEfSe) (Chang et al., 2022). The LDA threshold was chosen four and used All-against-all to evaluate the statistical differences. The variation of fungal community composition was analyzed by multiple group comparisons based on Welch’s *t*-test. The difference between plant biomass and soil properties were analyzed using the least significant difference (LSD) (Urbanová et al., 2015).

### 2.6. Network construction

The fungal network of plant treatments (PT) and plant combined with urban sludge treatments (PUT) were constructed on integrated network analysis based on random matrix theory (RMT) on iNAP platform (Feng K. et al., 2022). The OTUs level with abundance of >0.01 were filled based on Spearman’s rank correlation. Before the analysis, only OTUs detected in >20% of all samples were used for network construction. The largest and smallest cutoff value for RMT scanning was 1.0 and 0.01 separably. The module orders for each species were obtained by the fast-greedy method based on Spearman’s correlation. The nodes of fungi were assigned to the peripheral, connector, module hub, or network hub, according to their patterns of within- and among-module connections (Zi and Pi). The calculation was permuted by 100 times at each step of node removal at the proportion. Network properties, including node and edge number, connectedness, modularity, average path distance, and proportions of positive and negative edges were calculated and selected for comparison of networks. Module-Eigen Gene analysis was used to analyze the relationship between modules and environmental factors. The globe network of PT and PUT were visualized by Gephi 0.9.2 with the “Fruchterman Reingold” layout algorithm (Bastian et al., 2019).

## 3. Results

### 3.1. Soil properties and plant biomass

Soil properties were analyzed and compared in 14 treatments soil (Table 1). The content of AN and AP in plant treatments had no significant difference compared with untreated quartz tailings soil. Notably, AN and AP of WH, WZ, WHZ, WYHZ, and AN of WY were significantly higher than in untreated quartz tailings soil (*r* = 0.46, *P* < 0.05). The content of AN and AP were increased with the addition of urban sludge. pH value of quartz tailings soil was increased after all treatments. In addition, the content of Fe_2_O_3_ in KH, KZ, KYH, KYZ, KHZ, KYHZ, WH, WZ, and WYHZ was significantly higher than that in untreated soil (*P* < 0.05), while the content of SiO_2_ had no significant difference between treated and untreated tailings soil. The biomass of three plant species in plant combined with urban sludge treatments were higher than that in plant treatments (Supplementary Table 1) (*P* < 0.05). The highest biomass of Y, H and Z were in WYH, WYHZ, and WYZ, respectively. The total biomass of plant combined with urban sludge treatments was higher than in plant treatments (*P* < 0.05).

**TABLE 1:** Effect of plant and plant combined with urban sludge treatments on quartz tailings soil properties.

Treatment	Available nitrogen (AN, mg/kg)	Available phosphorus (AP, mg/kg)	Iron trioxide (Fe_2_O_3_, %)	Silicon dioxide (SiO_2_, %)	Total carbon (TC, %)	pH
Untreated soil	14.00 ± 2.13^d^	1.86 ± 0.15^bc^	1.25 ± 0.13^d^	52.83 ± 0.58^abc^	0	5.35 ± 0.21^f^
KY	10.62 ± 3.26^d^	1.93 ± 0.09^c^	1.82 ± 0.16^bcd^	51.46 ± 0.42^bc^	0	5.52 ± 0.06^ef^
KH	11.18 ± 2.08^d^	1.53 ± 0.05^c^	2.40 ± 0.26^ab^	52.79 ± 0.80^abc^	0	7.14 ± 0.32^a^
KZ	16.16 ± 1.79^d^	1.49 ± 0.08^c^	2.58 ± 0.06^a^	53.28 ± 1.15^ab^	0	6.58 ± 0.07^bc^
WY	35.64 ± 5.15^ab^	15.72 ± 1.29^b^	1.91 ± 0.09^bcd^	51.37 ± 1.20^bc^	0	5.44 ± 0.07^f^
WH	36.90 ± 4.12^a^	39.42 ± 3.53^a^	2.04 ± 0.28^abc^	50.90 ± 0.72^c^	0	6.42 ± 0.39^bcd^
WZ	43.53 ± 7.35^a^	40.17 ± 11.16^a^	2.00 ± 0.24^abc^	52.49 ± 0.90^abc^	0	6.00 ± 0.03^de^
KYH	12.59 ± 1.66^d^	1.41 ± 0.22^c^	2.57 ± 0.09^a^	53.08 ± 0.87^abc^	0	6.47 ± 0.13^bcd^
KYZ	21.53 ± 5.07^bcd^	0.79 ± 0.33^c^	2.53 ± 0.09^a^	52.47 ± 0.96^abc^	0	6.50 ± 0.10^bcd^
KHZ	19.43 ± 4.20^cd^	0.58 ± 0.28^c^	2.35 ± 0.15^ab^	53.30 ± 1.14^ab^	0	6.85 ± 0.22^ab^
WYH	15.32 ± 3.12^d^	8.78 ± 1.26^bc^	1.83 ± 0.23^bcd^	50.85 ± 0.60^c^	0	6.19 ± 0.11^cd^
WYZ	21.23 ± 0.78^bcd^	13.79 ± 0.60^bc^	1.74 ± 0.13^cd^	53.96 ± 0.86^a^	0	6.35 ± 0.09^bcd^
WHZ	31.85 ± 4.69^abc^	43.49 ± 10.13^a^	1.88 ± 0.39^bcd^	52.69 ± 0.71^abc^	0	7.15 ± 0.30^a^
KYHZ	13.56 ± 2.33^d^	1.55 ± 0.04^c^	2.30 ± 0.16^abc^	52.16 ± 0.43^abc^	0	6.43 ± 0.05^bcd^
WYHZ	42.55 ± 13.47^a^	38.96 ± 8.53^a^	2.37 ± 0.34^ab^	52.42 ± 0.39^abc^	0	6.49 ± 0.22^bcd^
Urban sludge	4111.33 ± 109.11	14.61 ± 0.38	3.25 ± 0.04	52.27 ± 0.41	0	6.03 ± 0.01

a, b, c, d, e, and f indicated significant differences (*P* < 0.05).

### 3.2. Effects of different treatments on fungal diversity and community composition

**FIGURE 2:**
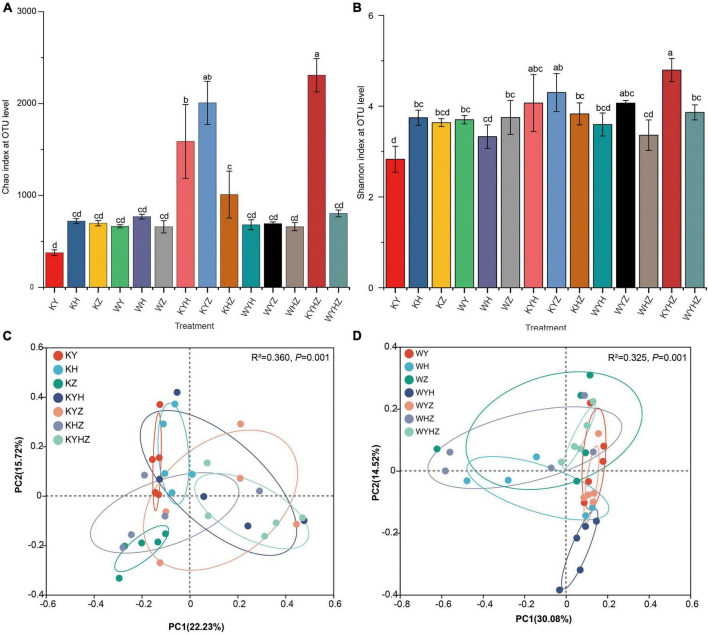
Chao index **(A)** and Shannon index **(B)** based on fungal OTUs level were analyzed in different treatments. Statistical differences between pairwise groups were determined using Student’s *t*-test. Panels **(A–D)** indicate significant differences (*P* < 0.05). Beta diversity of fungal community in plant treatments **(C)** and plant combined with urban sludge treatments **(D)** at the genus level was analyzed by principal coordinate (PCoA) with Adonis statistics based on Bray–Curtis distance.

The soil fungal alpha diversity was analyzed based on Chao and Shannon index (Figures 2A, B). The fungal alpha diversity in KY was the lowest compared with KH and KZ (*P* < 0.05). Interestingly, the fungal alpha diversity significantly increased under Y combined with other plant species in plant treatments. However, the addition of urban sludge significantly decreased the fungal alpha diversity in the plant species combination treatments (*P* < 0.05). Notably, fungal alpha diversity in WYHZ and WYZ were higher than other treatments, respectively. Soil fungal community composition of KY, KH, KZ, and KYHZ were separated from each other while that of KYH, KYZ, KHZ, and KYHZ cannot be separated with each other at the genus level (Figure 2C). The fungal community composition in WYH was different with other plant combined with urban sludge treatments (Figure 2D). Moreover, the plant treatments were separated from plant combined with urban sludge treatments.

**FIGURE 3:**
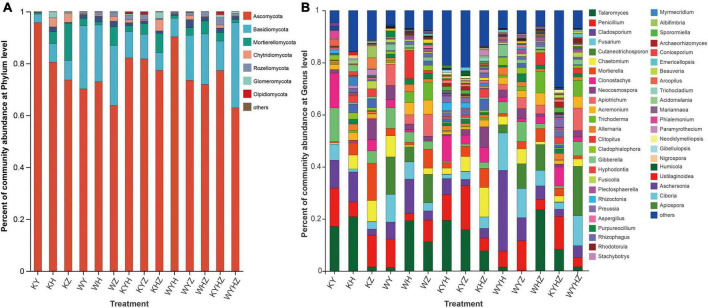
The abundance of taxa were displayed on the stacked column, and taxa with relative abundances less than 1% were combined into others. **(A)** At phylum level. **(B)** At the genus level.

**FIGURE 4:**
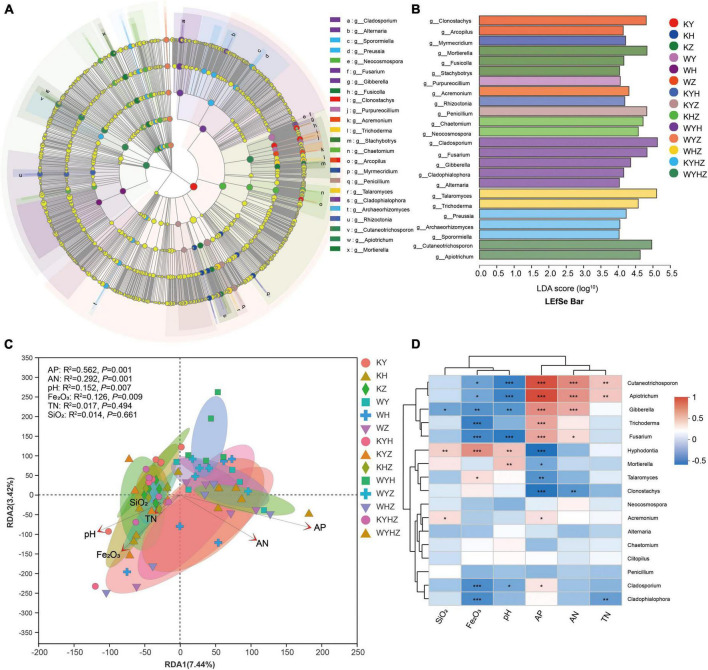
LEfSe analysis of the fungal abundance in different treatments. **(A)** The cladogram of fungal communities. **(B)** LDA score identified the size of differentiation of treatments with a threshold value of four. Relationship between soil fungal community composition and physicochemical properties revealed based on RDA analysis **(C)**. Arrows indicate the relationship between environmental factors and fungal profile. Spearman correlation heatmap of the top 20 genera and environmental factors **(D)**. *, ** and *** indicate *P* < 0.05, *P* < 0.01, *P* < 0.001, respectively.

The fungal community composition at the phylum and genus level were analyzed, Ascomycota, Basidiomycota, and Mortierellomycota were dominant phyla (Figure 3A). The addition of urban sludge decreased the abundance of Ascomycota and Mortierellomycota except for WYH, while increased the abundance of Basidiomycota. Additionally, the fungal community composition at the genus level among all treatments were significant difference (Figure 3B and Supplementary Figure 1). *Talaromyces*, *Penicillium*, *Clonostachys*, *Cladosporium*, *Mortierella*, *Chaetomium*, *Fusarium*, *Neocosmospora*, *Hyphodontia*, and *Acremonium* were dominant genera in plant treatments (Supplementary Figure 2A). However, the dominant genera in plant combined with urban sludge treatments were different with the plant treatments (Supplementary Figure 2B). The biomarkers in KYHZ and KZ were different with other plant treatments based on LEfSe analysis (LDA >4) (Figures 4A, B). *Mortierella*, *Fusicolla* and *Stachybotrys* in KZ were dominant. *Cladosporium*, *Fusarium*, *Gibberella*, *Cladophialophora*, and *Alternaria* in WYH were dominant genera. Notably, the abundance of *Cutaneotrichosporon* and *Apiotrichum* were significantly higher in WYHZ than in other treatments.

### 3.3. Effects of soil properties on fungal community composition

We analyzed the relationship between fungal community composition and soil properties using RDA analysis (Figure 4C). The results demonstrated that AP, AN, pH, and Fe_2_O_3_ significantly associated with the soil fungal community composition (*P* < 0.01) (Figure 4D). AP significantly affected on *Cutaneotrichosporon*, *Apiotrichum*, *Gibberella*, *Trichoderma*, *Fusarium*, *Acremonium*, *Cladosporium*, *Hyphodontia*, *Mortierella*, *Talaromyces* and *Clonostachys*, and had negative correlation with the latter four genera. AN had significant positive correlation with *Cutaneotrichosporon*, *Apiotrichum*, *Gibberella*, and *Fusarium*, but negative correlation with *Clonostachys*. pH had significant positive correlation with *Hyphodontia* and *Mortierella*, and negative correlation with *Cutaneotrichosporon*, *Apiotrichum*, *Gibberella*, *Fusarium*, and *Cladosporium*. Moreover, Fe_2_O_3_ had a positive correlation with *Cutaneotrichosporon*, *Apiotrichum*, *Gibberella*, *Trichoderma*, *Fusarium*, *Cladosporium*, and *Cladophialophoron*.

### 3.4. Fungal network analysis

**FIGURE 5:**
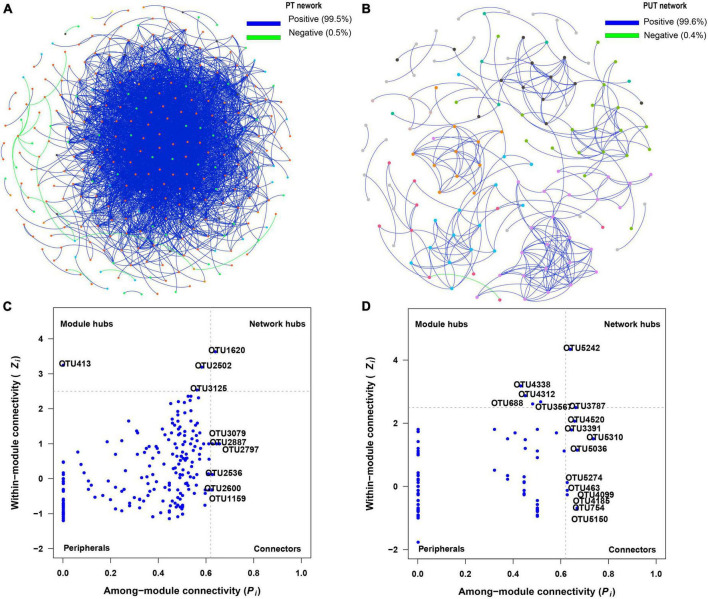
Visualization of fungal network of PT **(A)** and PUT **(B)**. The peripherals, connectors, module hubs, and network hubs are shown of PT **(C)** and PUT **(D)** fungal network. The colorful nodes represent the fungal OTUs. The blue edges indicate positive interaction between two individual nodes, while the green edges indicate negative interaction.

The results of the fungal network analysis showed that 3,574 and 277 edges were analyzed in the PT and PUT network, respectively (Table 2 and Figure 5A). PT network had higher total nodes, total links, connectedness, lower average path distance and modularity than PUT network. PT network was more complicated than PUT network. Furthermore, *Strelitziana* was network hub species, and *Didymella*, *Trichophaeopsis* were module hub species in PT network (Figure 5, Supplementary Figure 5, and Supplementary Table 2). *Fusarium* was network hub species, *Alternaria*, *Fusarium*, *Cerrena* were module hub species in PUT network. MEblue and MEbrown in PT network were negatively correlated with AP and AN, and positively correlated with pH (Supplementary Figure 3A). MEblue, MEbrown, MEsalmon, and MEgreen in PUT network were positively while MEyellow negatively correlated with AP andTN (Supplementary Figure 3B). MEblue was significantly correlated with MEbrown and MEyellow in PT network (Supplementary Figure 3C). MEbrown was significantly correlated with MEpurple, MEyellow, MEblack, and MEblue in PUT network. MEblue, MEpink, and MEmagenta in PUT network were positively while MEbalck and MEbrown negatively correlated with pH (Supplementary Figure 3D). MEblack and MEpurple in PUT network were positively correlated with AP, while MEpink was negatively correlated with AP (*P* < 0.05) (Supplementary Figure 3D). Key species in all modules were shown based on Module-EigenGene Analysis (Supplementary Figure 4).

**TABLE 2:** Topological characteristics of the basic parameters in PT and PUT fungal network.

Fungal network	Node number	Edge number	Connectedness	Modularity	Average path distance
PT	295	3574	0.78	0.17	2.51
PUT	131	277	0.13	0.81	2.61

PT, plant treatments; PUT, plant combined with urban sludge treatments.

## 4. Discussion

### 4.1. Effects of plant treatments on fungal diversity and community composition

In our study, soil fungal diversity in plant treatments significantly increased with plant species richness. The result supported our first hypothesis and consistent with previous research (Gil-Martínez et al., 2021). The higher richness of the plant species may increase resource quantity of soil, and then increase fungal available niches (Waldrop et al., 2006; Cline et al., 2018). Moreover, soil fungal community composition in plant treatments was strongly driven by plant species (Figure 2). Plant can recruit their own fungal community as decomposer, mutualists, and inhibiting pathogens (Schlechter et al., 2019; Vives-Peris et al., 2020; Santoyo, 2021). Ascomycota that was the dominant phyla in all treatments can degrade cellulose and more complex carbohydrates and adapt to nutrient-poor and dry habitats (Lin et al., 2019; Shen et al., 2022). For example, *Preussia* and Archaeorhizomyces which were dominated in our samples. *Preussia* can produced glucosidases, phosphatases, cellulases, and IAA to promote plant development under stressful environmental conditions (Kandar et al., 2018, Toppo et al., 2022). Archaeorhizomyces can enhance plant bioactive components, improve stress resistance, and alleviate the occurrence of diseases (Zhang et al., 2020). Furthermore, *Mortierella*, *Fusicolla*, and *Stachybotrys* play an essential role in reducing the effects of environmental stresses on plant growth, preventing disease and even promoting bioremediation (Zhang et al., 2016; Li et al., 2021). *Cutaneotrichosporon* and *Apiotrichum* belonging to Basidiomycota were potential antagonist of soil-borne plant pathogens and plant growth promoter (Ortega et al., 2020; Buratti et al., 2022). Furthermore, *Apiotrichum* may degrade heavy metal pollution, and promote plant growth (Yalçın et al., 2018; Kumla et al., 2020; Xie et al., 2021). The results indicated that plant indirectly or directly promoted the fungal diversity and adopt the stressful environment of tailing soil through recruiting potential beneficial fungal community (Wilkinson et al., 2019; Mattoo and Non-zom, 2021).

### 4.2. Variation and drivers of fungal community under plant combined with urban sludge treatments

In this study, soil properties, such as AN, AP, pH, TN, and Fe_2_O_3_ significantly affect soil fungal community composition. Furthermore, the addition of urban sludge significantly increased the content of AN and AP while decreased the fungal diversity compared with plant treatments (Figure 2). The results suggested that AN and AP are the essential drivers in explaining the changes of fungal diversity, especially for acidic soil. Soil AN and TN mainly came from urban sludge and N-fixation of Legumes in 14 treatments. Our results showed that *Cutaneotrichosporon* and *Apiotrichum* were significantly associated with AN and TN. AN enrichment decreased soil fungal diversity due to toxic of more protons or weaken the linkage between soil carbon and fungal diversity (Yang et al., 2022c). Moreover, AN enrichment might aggravate the water limitation by promoting plant growth that were proved by the increase of plant biomass according our results. The water limitation may decrease some fungal survive (Angel et al., 2010). Moreover, AP positively and significantly associated with *Cutaneotrichosporon*, *Apiotrichum*, *Trichodema*, and *Gibberella* that were dominated in treatments. The results suggested that AP is important driver for fungal community composition (Bulgarelli et al., 2022).

Soil pH directly or indirectly effects fungal community composition in many ecosystems (Shen et al., 2020; Queiroz et al., 2021). Tailings soil with low pH is toxic for plant or soil microbial community. Our data showed that pH was increased after plant or plant combined with urban sludge treatments. The results suggested that pH was the crucial predictor for fungal community composition. After all, less fungal community can survive in lower pH soil. Thus, plant or organic matters in urban sludge mitigated soil acidification, and led to change of fungal community. Moreover, pH and Fe_2_O_3_ negatively and significantly associated with *Fusarium* indicated that can inhibit pathogen. Notably, the content of Fe_2_O_3_ increased after plant or plant combined with urban sludge treatments and then enhance plant biomass (Feng Y. et al., 2022). This may be related to that the increase of pH is conducive to the dissociation of iron oxides (Penn and Camberato, 2019). Notably, some fungi, such as *Talaromyces*, *Penicillium*, and *Cladosporium* can produce siderophores and then increase the content of Fe_2_O_3_ (Crowley, 2006; Pourhassan et al., 2014; Sahu and Prakash, 2021). Together, our findings on the changes of fungal community and soil properties after treatments supported that plant or plant combined with urban sludge effectively improve environment of tailings soil.

### 4.3. Fungal network under plant and plant combined with urban sludge treatments

In this study, the PT network had more node, edge numbers and complexity than the PUT network (Figure 5 and Table 2). More network connectors make the links stronger and make the network more stable. *Strelitziana* was network hub species, and *Didymella*, *Trichophaeopsis* were module hub species in PT network. The hub species were saprotrophs and mycorrhizal fungi that enhance plant nutrient uptake and development. AN was important driver of modular structure in PT fungal network. This suggests that fungal community tends to cooperate with each other to resist stress. Fungi-fungi feedback under low soil nutrition increases the stability and complexity of networks (Yang et al., 2022a). Furthermore, the addition of urban sludge increased modularity of network. In general, high modularity in the network was more beneficial to increase asynchronism, thus reducing the influence of species loss on the overall network (Yang et al., 2022b). Moreover, the addition of urban sludge changed the soil properties, and AP, TN, pH, AN, and Fe_2_O_3_ significantly drive modular structure in PUT fungal network. Notably, *Fusarium* that are usually recognized as plant pathogens was hub species in PUT network. *Fusarium* has strong competitive ability by mycotoxin production (Karlsson et al., 2021). This suggests that pathogens may suppress other fungal groups and led to the decrease of stability and complexity of fungal network in PUT network.

## 5. Conclusion

In this study, we analyzed the fungal diversity and community composition under plant and plant combined with urban sludge treatments to remediate quartz tailings soil. The results suggest that plant diversity determine soil fungal diversity. Plant or plant combined with urban sludge significantly moderate acidification condition and increase nutrients of tailings soil. Plant can recruit beneficial fungi to improve their development and assist resist stress. Effectively remediate tailings soil due to the improvement of biological-chemical-physical properties. Fungal network is more stable and complex in plant treatments while fungal network is more fragile with the addition of urban sludge. AN and AP enrichment in urban sludge may be important drivers for fungal network. Future work should address the functional characters of fungal community when tailings soil is remediated by plant or urban sludge. Isolation and application of fungal functional groups are vital for remediation of tailings soil.

## Data availability statement

The datasets presented in this study can be found in online repositories. The names of the repository/repositories and accession number(s) can be found in this article/Supplementary material.

## Author contributions

XL conceived and designed the experiments. FD analyzed the data and wrote the manuscript. YZ, XZ, and CZ analyzed the data. TS, YT, and YW conducted the pot experiments and measured soil parameters and plant biomass. All authors contributed to the article and approved the submitted version.

## References

[B1] Adiansyah J. S. Rosano M. Vink S. Keir G. (2015). A framework for a sustainable approach to mine tailings management: disposal strategies. *J. Clea. Prod.* 108 1050–1062. 10.1016/j.jclepro.2015.07.139

[B2] Alcantara H. J. P. Doronila A. I. Nicolas M. Ebbs S. D. Kolev S. D. (2015). Growth of selected plant species in biosolids-amended mine tailings. *Min. Eng.* 80 25–32. 10.1016/j.mineng.2015.06.012

[B3] Ali S. Abbas Z. Rizwan M. Zaheer I. Yavaş I. Ünay A. (2020). Application of floating aquatic plants in phytoremediation of heavy metals polluted water: a review. *Sustainability* 12:1927. 10.3390/su12051927

[B4] Angel R. M. Ines M. S. Eugene D. U. Osnat G. (2010). “Biogeography of soil archaea and bacteria along a steep precipitation gradient”. *ISME J.* 4 553–563. 10.1038/ismej.2009.136 20033070

[B5] Arthur E. L. Rice P. J. Rice P. J. Anderson T. A. Baladi S. M. Henderson K. L. D. (2005). Phytoremediation—an overview. *Crit. Rev. Plant Sci.* 24 109–122. 10.1080/07352680590952496

[B6] Asensio V. Vega F. A. Andrade M. L. Covelo E. F. (2013a). Technosols made of wastes to improve physico-chemical characteristics of a copper mine soil. *Pedosphere* 23 1–9. 10.1016/S1002-0160(12)60074-5

[B7] Asensio V. Vega F. A. Singh B. R. Covelo E. F. (2013b). Effects of tree vegetation and waste amendments on the fractionation of Cr, Cu, Ni, Pb and Zn in polluted mine soils. *Sci. Total Environ.* 443 446–453. 10.1016/j.scitotenv.2012.09.069 23220134

[B8] Bai Y. Mei L. Zuo W. Zhang Y. Gu C. Shan Y. (2019). Response of bacterial communities in coastal mudflat saline soil to sewage sludge amendment. *Appl. Soil Ecol.* 144 107–111. 10.1016/j.apsoil.2019.07.007

[B9] Bao Y. Su D. Yang W. Zhao Y. (2022). Study on soil remediation effect of copper tailings pond and surrounding vegetation restoration model. *Multi. Utilizati. Min. Res.* 2022 74–81.

[B10] Bastian M. Heymann S. Jacomy M. (2019). “Gephi: an open source software for exploring and manipulating networks,” in *Proceedings of the international AAAI conference on web and social media*, 361–362.

[B11] Begum N. Ahanger M. A. Su Y. Lei Y. Mustafa N. S. A. Ahmad P. (2019). Improved drought tolerance by AMF inoculation in maize (Zea mays) involves physiological and biochemical implications. *Plants* 8 579. 10.3390/plants8120579 31817760 PMC6963921

[B12] Bukhori M. F. M. Ketol B. Razali K. R. Hussain A. Rohmon M. F. (2020). Brief documentation of basidiomycota and ascomycota diversity in gunung gading national park, sarawak. *J. Sci. Mathe. Lett.* 8 37–47.

[B13] Bulgarelli R. G. Leite M. F. A. de Hollander M. Mazzafera P. Andrade S. A. L. Kuramae E. E. (2022). Eucalypt species drive rhizosphere bacterial and fungal community assembly but soil phosphorus availability rearranges the microbiome. *Sci. Total Environ.* 836:155667. 10.1016/j.scitotenv.2022.155667 35513142

[B14] Buratti S. Girometta C. E. Baiguera R. M. Barucco B. Bernardi M. De Girolamo G. (2022). Fungal diversity in two wastewater treatment plants in north Italy. *Microorganisms* 10:1096. 10.3390/microorganisms10061096 35744613 PMC9229248

[B15] Capblancq T. Forester B. R. (2021). Redundancy analysis: a swiss army knife for landscape genomics. *Methods Ecol. Evolut.* 12 2298–2309.

[B16] Caporaso J. G. Kuczynski J. Stombaugh J. Bittinger K. Bushman F. D. Costello E. K. (2010). QIIME allows analysis of high-throughput community sequencing data. *Nat. Methods* 7 335–336. 20383131 10.1038/nmeth.f.303 PMC3156573

[B17] Carvalho F. P. (2017). Mining industry and sustainable development: time for change. *Food Energy Security* 6 61–77. 10.1002/fes3.109

[B18] Chandra R. Kumar V. (2018). Phytoremediation: a green sustainable technology for industrial waste management. *Phytoremed. Environ. Pollut.* CRC Press, 1–42.

[B19] Chang F. He S. Dang C. (2022). Assisted selection of biomarkers by linear discriminant analysis effect size (lefse) in microbiome data. *J. Visu. Exp.* 2022:183. 10.3791/61715 35635468

[B20] Cheng K. Khokhar M. S. Ayoub M. Jamali Z. (2021). Nonlinear dimensionality reduction in robot vision for industrial monitoring process via deep three dimensional Spearman correlation analysis (D3D-SCA). *Multi. Tools Appli.* 80 5997–6017.

[B21] Cline L. C. Hobbie S. E. Madritch M. D. Buyarski C. R. Tilman D. Cavender-Bares J. M. (2018). Resource availability underlies the plant-fungal diversity relationship in a grassland ecosystem. *Ecology* 99 204–216. 10.1002/ecy.2075 29106700

[B22] Crowley D. E. (2006). Microbial siderophores in the plant rhizosphere. *Iron Nutr. Plants Rhizos. Microorg.* 2006 169–198.

[B23] da Silva A. P. V. Silva A. O. Lima F. R. D. Benedet L. Franco A. J. Souza J. K. (2022). Potentially toxic elements in iron mine tailings: effects of reducing soil pH on available concentrations of toxic elements. *Environ. Res.* 215:114321. 10.1016/j.envres.2022.114321 36222244

[B24] Debeljak M. van Elteren J. T. Špruk A. Izmer A. Vanhaecke F. Vogel-Mikuš K. (2018). The role of arbuscular mycorrhiza in mercury and mineral nutrient uptake in maize. *Chemosphere* 212 1076–1084. 10.1016/j.chemosphere.2018.08.147 30286537

[B25] Dickinson N. (2017). “Phytoremediation,” in *Encyclopedia of applied plant sciences*, eds Thomas B. Murray B. G. Murphy D. J. (Oxford: Academic Press), 327–331.

[B26] Edgar R. C. (2013). UPARSE: highly accurate OTU sequences from microbial amplicon reads. *Nat. Methods* 10 996–998. 10.1038/nmeth.2604 23955772

[B27] Elfiati D. Susilowati A. Siagian T. M. (2021). “Characterization of phosphate solubilizing and cellulolytic fungi isolated from soil under eurycoma longifolia stands,” in *Proceeding of the IOP conference series: earth and environmental science*, 10.1088/1755-1315/886/1/012018

[B28] Faucon M. P. Shutcha M. N. Meerts P. (2007). Revisiting copper and cobalt concentrations in supposed hyperaccumulators from SC Africa: influence of washing and metal concentrations in soil. *Plant and Soil* 301, 29–36. 10.1007/s11104-007-9405-3

[B29] Feng K. Peng X. Zhang Z. Gu S. He Q. Shen W. (2022). iNAP: an integrated network analysis pipeline for microbiome studies. *iMeta* 1:13. 10.1002/imt2.13 PMC10989900 38868563

[B30] Feng Y. Kreslavski V. D. Shmarev A. N. Ivanov A. A. Zharmukhamedov S. K. Kosobryukhov A. (2022). Effects of iron oxide nanoparticles (Fe3O4) on growth, photosynthesis, antioxidant activity and distribution of mineral elements in wheat (*Triticum aestivum*) Plants. *Plants* 11:1894. 10.3390/plants11141894 35890527 PMC9322615

[B31] Festin E. S. Tigabu M. Chileshe M. N. Syampungani S. Odén P. C. (2018). Progresses in restoration of post-mining landscape in Africa. *J. Forest. Res.* 30 381–396. 10.1007/s11676-018-0621-x

[B32] Frac M. Hannula S. E. Belka M. Jedryczka M. (2018). Fungal biodiversity and their role in soil health. *Front. Microbiol*. 9, 707. 10.3389/fmicb.2018.00707 29755421 PMC5932366

[B33] Fuke P. Kumar M. Sawarkar A. D. Pandey A. Singh L. (2021). Role of microbial diversity to influence the growth and environmental remediation capacity of bamboo: a review. *Industr. Crops Prod.* 167:113567. 10.1016/j.indcrop.2021.113567

[B34] Geetha N. Sunilkumar C. R. Bhavya G. Nandini B. Abhijith P. Satapute P. (2022). Warhorses in soil bioremediation: seed biopriming with PGPF secretome to phytostimulate crop health under heavy metal stress. *Environ. Res.* 2022:114498. 10.1016/j.envres.2022.114498 36209791

[B35] Gil-Martínez M. López-García Á Domínguez M. T. Kjøller R. Navarro-Fernández C. M. Rosendahl S. (2021). Soil fungal diversity and functionality are driven by plant species used in phytoremediation. *Soil Biol. Biochem.* 153:108102. 10.1016/j.soilbio.2020.108102

[B36] Ikram M. Ali N. Jan G. Jan F. G. Rahman I. U. Iqbal A. (2018). IAA producing fungal endophyte *Penicillium roqueforti* thom, enhances stress tolerance and nutrients uptake in wheat plants grown on heavy metal contaminated soils. *PLoS One* 13:e0208150. 10.1371/journal.pone.0208150 30496253 PMC6264496

[B37] Irhema S. I. S. (2019). *Feasibility of soil amendments and ryegrass in circum-neutral and acidic mine tailings remediation.* Bangor University (United Kingdom).

[B38] Jia T. Wang R. Chai B. (2019). Effects of heavy metal pollution on soil physicochemical properties and microbial diversity over different reclamation years in a copper tailings dam. *J. Soil Water Conservat.* 74 439–448. 10.2489/jswc.74.5.439

[B39] Kandar M. Suhandono S. Aryantha I. N. P. (2018). Growth promotion of rice plant by endophytic fungi. *J. Pure Appl. Microbiol.* 12 1569–1577.

[B40] Karlsson I. Persson P. Friberg H. (2021). Fusarium head blight from a microbiome perspective. *Front. Microbiol.* 12:628373. 10.3389/fmicb.2021.628373 33732223 PMC7956947

[B41] Kembel S. W. Cowan P. D. Helmus M. R. Cornwell W. K. Morlon H. Ackerly D. D. (2010). Picante: R tools for integrating phylogenies and ecology. *Bioinformatics* 26 1463–1464. 10.1093/bioinformatics/btq166 20395285

[B42] Kossoff D. Dubbin W. E. Alfredsson M. Edwards S. J. Macklin M. G. Hudson-Edwards K. A. (2014). Mine tailings dams: characteristics, failure, environmental impacts, and remediation. *Appl. Geochem.* 51 229–245. 10.1016/j.apgeochem.2014.09.010

[B43] Kumla J. Nundaeng S. Suwannarach N. Lumyong S. (2020). Evaluation of multifarious plant growth promoting trials of yeast isolated from the soil of assam tea (camellia sinensis var. assamica) plantations in Northern Thailand. *Microorganisms* 8:1168. 10.3390/microorganisms8081168 32752164 PMC7465209

[B44] Li W. Long Y. Mo F. Shu R. Yin X. Wu X. (2021). Antifungal activity and biocontrol mechanism of *Fusicolla violacea* J-1 against soft rot in kiwifruit caused by alternaria alternata. *J. Fungi* 7:937. 10.3390/jof7110937 34829224 PMC8620048

[B45] Lin Y. Ye Y. Hu Y. Shi H. (2019). The variation in microbial community structure under different heavy metal contamination levels in paddy soils. *Ecotoxicol. Environ. Safety* 180 557–564. 10.1016/j.ecoenv.2019.05.057 31128554

[B46] Mangan S. A. Schnitzer S. A. Herre E. A. Mack K. M. L. Valencia M. C. Sanchez E. I. (2010). Negative plant–soil feedback predicts tree-species relative abundance in a tropical forest. *Nature* 466 752–755. 10.1038/nature09273 20581819

[B47] Mattoo A. J. Non-zom S. (2021). Endophytic fungi: understanding complex cross-talks. *Symbiosis* 83 237–264. 10.1007/s13199-020-00744-2

[B48] Ortega H. E. Torres-Mendoza D. Cubilla-Rios L. (2020). Patents on endophytic fungi for agriculture and bio- and phytoremediation applications. *Microorganisms* 8:1237. 10.3390/microorganisms8081237 32823804 PMC7465599

[B49] Ozimek E. Hanaka A. (2020). Mortierella species as the plant growth-promoting fungi present in the agricultural soils. *Agriculture* 11:10007. 10.3390/agriculture11010007

[B50] Penn C. J. Camberato J. J. (2019). A critical review on soil chemical processes that control how soil pH affects phosphorus availability to plants. *Agriculture* 9:120. 10.1016/j.biotechadv.2009.02.006 19269313

[B51] Pourhassan N. Gagnon R. Wichard T. Bellenger J.-P. (2014). Identification of the hydroxamate siderophore ferricrocin in Cladosporium cladosporioides. *Nat. Prod. Commun.* 9:1934578X1400900429. 24868878

[B52] Qi D. Wieneke X. Zhou X. Jiang X. Xue P. (2017). Succession of plant community composition and leaf functional traits in responding to karst rocky desertification in the Wushan County in Chongqing, China. *Commun. Ecol.* 18 157–168. 10.1556/168.2017.18.2.5

[B53] Queiroz M. E. F. Monteiro J. S. Viana-Junior A. B. Praxedes C. L. B. Lavelle P. Vasconcelos S. S. (2021). Litter thickness and soil pH influence the diversity of saprotrophic fungi in primary forest fragments in the Amazon. *Pedobiologia* 89:150771. 10.1016/j.pedobi.2021.150771

[B54] Sahu S. Prakash A. (2021). Siderophore from talaromyces trachyspermus: augmentation and characterization. *bioRxiv* [preprint]. bioRxiv:2021.2004. 2013.439607.

[B55] Sanchez V. Lopez-Bellido F. J. Canizares P. Rodriguez L. (2018). Can electrochemistry enhance the removal of organic pollutants by phytoremediation? *J. Environ. Manage.* 225 280–287. 10.1016/j.jenvman.2018.07.086 30098494

[B56] Santibañez C. de la Fuente L. M. Bustamante E. Silva S. León-Lobos P. Ginocchio R. (2012). Potential use of organic- and hard-rock mine wastes on aided phytostabilization of large-scale mine tailings under semiarid mediterranean climatic conditions: short-term field study. *Appl. Environ. Soil Sci.* 2012:895817. 10.1155/2012/895817

[B57] Santoyo G. (2021). How plants recruit their microbiome? New insights into beneficial interactions. *J. Adv. Res.* 40 45–58. 10.1016/j.jare.2021.11.020 36100333 PMC9481936

[B58] Schlechter R. O. Miebach M. Remus-Emsermann M. N. (2019). Driving factors of epiphytic bacterial communities: a review. *J. Adv. Res.* 19 57–65. 31341670 10.1016/j.jare.2019.03.003 PMC6630024

[B59] Sharma I. (2020). “Bioremediation techniques for polluted environment: concept, advantages, limitations, and prospects,” in *Trace metals in the environment-new approaches and recent advances*, (IntechOpen), eds Mario Alfonso Murillo-Tovar, Hugo Saldarriaga-Noreña and Agnieszka Saeid.

[B60] Shen C. Gunina A. Luo Y. Wang J. He J. Z. Kuzyakov Y. (2020). Contrasting patterns and drivers of soil bacterial and fungal diversity across a mountain gradient. *Environ. Microbiol.* 22 3287–3301. 32436332 10.1111/1462-2920.15090

[B61] Shen Y. Li H. Liu Y. Gao T. Li G. Zuo M. (2022). Variations of fungal communities in lead–zinc tailings located in Northwestern China. *Hum. Ecol. Risk Assess. Int. J.* 2022 1–20.

[B62] Sheoran A. Sheoran V. Poonia P. (2008). Rehabilitation of mine degraded land by metallophytes. *Mining Eng. J.* 10 11–16. 10.1016/j.scitotenv.2021.150659 34597555

[B63] Su R. Wang Y. Huang S. Chen R. Wang J. (2022). Application for ecological restoration of contaminated soil: phytoremediation. *Int. J. Environ. Res. Public Health* 19:124. 10.3390/ijerph192013124 36293698 PMC9603173

[B64] Sun A. Jiao X.-Y. Chen Q. Wu A.-L. Zheng Y. Lin Y.-X. (2021). Microbial communities in crop phyllosphere and root endosphere are more resistant than soil microbiota to fertilization. *Soil. Biol. Biochem.* 153:108113. 10.1016/j.soilbio.2020.108113

[B65] Sun R. Zhang X.-X. Guo X. Wang D. Chu H. (2015). Bacterial diversity in soils subjected to long-term chemical fertilization can be more stably maintained with the addition of livestock manure than wheat straw. *Soil Biol. Biochem.* 88 9–18. 10.1016/j.soilbio.2015.05.007

[B66] Templeton B. (2018). *Survey of canadian native plant species for resistance to salt and metal stress.* University of Saskatchewan.

[B67] Toppo P. Subba R. Roy K. Mukherjee S. Mathur P. (2022). Elucidating the strategies for isolation of endophytic fungi and their functional attributes for the regulation of plant growth and resilience to stress. *J. Plant Growth Regulat.* 2022 1–22.

[B68] Urbanová M. Šnajdr J. Baldrian P. (2015). Composition of fungal and bacterial communities in forest litter and soil is largely determined by dominant trees. *Soil Biol. Biochem.* 84 53–64. 10.1016/j.soilbio.2015.02.011

[B69] Villanueva R. A. M. Chen Z. J. (2019). ggplot2: elegant graphics for data analysis (2nd ed.). *Measurement: Interdisciplinary Research and Perspectives* 17, 160–167. 10.1080/15366367.2019.1565254

[B70] Vives-Peris V. de Ollas C. Gómez-Cadenas A. Pérez-Clemente R. M. (2020). Root exudates: from plant to rhizosphere and beyond. *Plant Cell Rep.* 39 3–17. 31346716 10.1007/s00299-019-02447-5

[B71] Waldrop M. P. Zak D. R. Blackwood C. B. Curtis C. D. Tilman D. (2006). Resource availability controls fungal diversity across a plant diversity gradient. *Ecol. Lett.* 9 1127–1135. 10.1111/j.1461-0248.2006.00965.x 16972876

[B72] Wang L. Ji B. Hu Y. Liu R. Sun W. (2017). A review on in situ phytoremediation of mine tailings. *Chemosphere* 184 594–600. 10.1016/j.chemosphere.2017.06.025 28623832

[B73] Wang Q.-Y. Liu J.-S. Wang Y. Yu H.-W. (2015). Accumulations of copper in apple orchard soils: distribution and availability in soil aggregate fractions. *J. Soils Sedim.* 15 1075–1082. 10.1007/s11368-015-1065-y

[B74] Wilkinson S. W. Magerøy M. H. López Sánchez A. Smith L. M. Furci L. Cotton T. E. A. (2019). Surviving in a hostile world: plant strategies to resist pests and diseases. *Annu Rev. Phytopathol.* 57 505–529. 10.1146/annurev-phyto-082718-095959 31470772

[B75] Wu B. Luo S. Luo H. Huang H. Xu F. Feng S. (2022). Improved phytoremediation of heavy metal contaminated soils by *Miscanthus floridulus* under a varied rhizosphere ecological characteristic. *Sci. Total Environ.* 808:151995. 10.1016/j.scitotenv.2021.151995 34856269

[B76] Xie Y. Bu H. Feng Q. Wassie M. Amee M. Jiang Y. (2021). Identification of Cd-resistant microorganisms from heavy metal-contaminated soil and its potential in promoting the growth and Cd accumulation of bermudagrass. *Environ. Res.* 200:111730. 10.1016/j.envres.2021.111730 34293315

[B77] Yalçın H. T. Ergin-Tepebaşı G. Uyar E. (2018). Isolation and molecular characterization of biosurfactant producing yeasts from the soil samples contaminated with petroleum derivatives. *J. Basic Microbiol.* 58 782–792. 10.1002/jobm.201800126 29938807

[B78] Yang T. Tedersoo L. Liu X. Gao G. F. Dong K. Adams J. M. (2022a). Fungi stabilize multi-kingdom community in a high elevation timberline ecosystem. *iMeta* 1:49. 10.1002/imt2.49 PMC10989762 38867896

[B79] Yang T. Tedersoo L. Soltis P. S. Soltis D. E. Sun M. Ma Y. (2022b). Plant and fungal species interactions differ between aboveground and belowground habitats in mountain forests of eastern China. *Sci. China Life Sci.* 2022:2174. 10.1007/s11427-022-2174-3 36462107

[B80] Yang Y. Chen X. Liu L. Li T. Dou Y. Qiao J. (2022c). Nitrogen fertilization weakens the linkage between soil carbon and microbial diversity: a global meta-analysis. *Global Change Biol.* 28 6446–6461. 10.1111/gcb.16361 35971768

[B81] Young I. Renault S. Markham J. (2015). Low levels organic amendments improve fertility and plant cover on non-acid generating gold mine tailings. *Ecol. Eng.* 74 250–257. 10.1016/j.ecoleng.2014.10.026

[B82] Yu H. Y. Wang X. Li F. Li B. Liu C. Wang Q. (2017). Arsenic mobility and bioavailability in paddy soil under iron compound amendments at different growth stages of rice. *Environ. Pollut.* 224 136–147. 10.1016/j.envpol.2017.01.072 28202263

[B83] Zhang C. B. Ren C. H. Wang Y. L. Wang Q. Q. Wang Y. S. Weng Q. B. (2020). Uncovering fungal community composition in natural habitat of *Ophiocordyceps sinensis* using high-throughput sequencing and culture-dependent approaches. *BMC Microbiol.* 20:331. 10.1186/s12866-020-01994-2 33138775 PMC7607863

[B84] Zhang W. Cao J. Zhang S. Wang C. (2016). Effect of earthworms and arbuscular mycorrhizal fungi on the microbial community and maize growth under salt stress. *Appl. Soil Ecol.* 107 214–223. 10.1016/j.apsoil.2016.06.005

[B85] Zhao A. Gao L. Chen B. Feng L. (2019). Phytoremediation potential of *Miscanthus sinensis* for mercury-polluted sites and its impacts on soil microbial community. *Environ. Sci. Pollut. Res. Int.* 26 34818–34829. 10.1007/s11356-019-06563-3 31654309

[B86] Zhao S. Liu M. Li H. Zeng Q. (2019). Comparison between pyrophosphate method and infrared spectrophotometry for determination of silicon dioxide. *Chin. J. Industr. Hygiene Occupat. Dis.* 37 781–784. 10.3760/cma.j.issn.1001-9391.2019.10.014 31726512

[B87] Zuo W. Gu C. Zhang W. Xu K. Wang Y. Bai Y. (2019). Sewage sludge amendment improved soil properties and sweet sorghum yield and quality in a newly reclaimed mudflat land. *Sci. Total Environ.* 654 541–549. 10.1016/j.scitotenv.2018.11.127 30447593

