# Corrigendum: Fungal community remediate quartz tailings soil under plant combined with urban sludge treatments

**DOI:** 10.3389/fmicb.2023.1265591

**Published:** 2024-01-15

**Authors:** Fabao Dong, Yujia Zhu, Xunmei Zhu, Chengzhi Zhang, Yingying Tao, Taotao Shao, Yue Wang, Xia Luo

**Affiliations:** ^1^School of Biological Science and Food Engineering, Chuzhou University, Chuzhou, Anhui, China; ^2^Center of Excellence in Fungal Research, Mae Fah Luang University, Chiang Rai, Thailand; ^3^School of Science, Mae Fah Luang University, Chiang Rai, Thailand; ^4^College of Food and Pharmaceutical Engineering, Guizhou Institute of Technology, Guiyang, Guizhou, China

**Keywords:** fungal diversity, fungal community structure, phytoremediation, quartz tailings, phosphorus and nitrogen, urban sludge

In the published article, there was an error in affiliation 1. Instead of “Center of Excellence in Fungal Research, Mae Fah Luang University, Chiang Rai, Thailand”, it should be “School of Biological Science and Food Engineering, Chuzhou University, Chuzhou, Anhui, China”.

In the published article, there was an error in affiliation 4. Instead of “School of Biological Science and Food Engineering, Chuzhou University, Chuzhou, Anhui, China”, it should be “College of Food and Pharmaceutical Engineering, Guizhou Institute of Technology, Guiyang, Guizhou, China”.

The authors apologize for this error and state that this does not change the scientific conclusions of the article in any way. The original article has been updated.

