# Supplementary material for: Fungal community remediate quartz tailings soil under plant combined with urban sludge treatments
Source: Front Microbiol. 2023 Apr 20;14:1160960. doi: 10.3389/fmicb.2023.1160960 (PMC10157048; doi:10.3389/fmicb.2023.1160960)
Supplement: Supplementary file 1 [file Data_Sheet_1.PDF]

Supplementary Material

Fungal community remediate quartz tailings soil under plant combined with urban sludge treatments

Fabao Dong, Yujia Zhu, Xunmei Zhu, Chengzhi Zhang, Taotao Shao, Yue Wang, Yingying Tao, Xia Luo*

*** Correspondence:**

Corresponding Author:

Xia Luo

luoxiacz@163.com

1 Supplementary Data

1.1 Supplementary Table

Table S1 Effect of plant and plant combined with urban sludge treatments on plant biomass

Plant species combination	Treatments	Dry weight of overground (g)	Dry weight of underground(g)	Total dry weight(g)	Overground length (cm)	Underground length(cm)	Total length(cm)
Y	KY	0.03273±0.00376d	0.0085±0.00116a	0.04123±0.00427c	21.25±1.22878d	5.54667±0.58504ab	26.79667±1.29137c
	WY	0.0717±0.00589ab	0.00547±0.000680995bc	0.07717±0.00614a	29.99333±1.51719a	5.03333±0.4178ab	35.02667±1.65331a
	KYH	0.03267±0.00437d	0.00593±0.000635959bc	0.0386±0.00469c	22.21333±1.35428cd	6.2±0.34282a	28.41333±1.38927bc
	WYH	0.07293±0.00561ab	0.0064±0.000888015ab	0.07933±0.00575a	27.38±1.16407ab	5.67333±0.45539ab	33.05333±1.25177ab
	KYZ	0.04607±0.00724cd	0.003±0.00039036cd	0.04907±0.00752bc	22.37333±1.1218cd	3.30667±0.27106c	25.68±1.20068c
	WYZ	0.08347±0.00864a	0.00193±0.000492483d	0.0854±0.00873a	31.69333±2.21775a	4.72±0.42731abc	36.41333±2.41936a
	KYHZ	0.0606±0.00877bc	0.0047±0.00148bcd	0.0653±0.00863ab	24.82±2.0923bcd	4.02±0.43609bc	28.84±2.2115bc
	WYHZ	0.0841±0.0121ab	0.0035±0.000909823bcd	0.0876±0.01238a	26.55±2.14643abc	3.19±0.62795c	29.74±2.30445bc
H	KH	0.05977±0.00743d	0.05687±0.01059c	0.11663±0.01647d	26.01±0.82892c	17.82667±0.99892bc	43.83667±1.52363d
	WH	0.38623±0.0249c	0.22253±0.03456b	0.60877±0.04902c	45.93667±1.11132a	16.44±0.90068c	62.37667±1.39712b
	KHZ	0.09433±0.0178d	0.04713±0.01984c	0.14147±0.03642d	29.3±1.33606bc	16.86±1.14359c	46.16±2.12645cd
	WHZ	0.6±0.07663b	0.43653±0.07893a	1.03653±0.11597b	49.37333±0.89344a	21.68667±1.40803a	71.06±1.94213a
	KYH	0.08847±0.01005d	0.04347±0.00753c	0.13193±0.01637d	32.50667±1.30232b	14.89333±1.57191c	47.4±2.64213cd
	WYH	0.5188±0.02788b	0.2358±0.0201b	0.7546±0.03908c	48.45333±1.43061a	18.74±0.92874abc	67.19333±1.77226ab
	KYHZ	0.1166±0.02d	0.0675±0.01167c	0.1841±0.0275d	33.16±2.65373b	17.96±2.16673abc	51.12±3.58589c
	WYHZ	0.7812±0.11788a	0.5094±0.12271a	1.2906±0.22005a	46.76±2.27704a	21.56±2.01771ab	68.32±3.68332ab
Z	KZ	0.01407±0.00131c	0.0037±0.000404429b	0.01777±0.00141c	7.06667±0.63823cd	7.28667±0.45609bc	14.35333±0.84305c
	WZ	0.10273±0.01681b	0.01707±0.0037a	0.1198±0.02025b	18.06±1.65624b	10.09667±0.59406b	28.15667±2.09338b
	KHZ	0.00587±0.00108c	0.00267±0.000360775b	0.00853±0.00134c	4.43333±0.5284cd	7.14±0.67934bc	11.57333±0.92667cd
	WHZ	0.01127±0.00242c	0.00647±0.00141b	0.01773±0.0037c	5.97333±0.63692cd	10.29333±0.79297b	16.26667±1.22212c
	KYZ	0.00827±0.00229c	0.00173±0.000462567b	0.01±0.00258c	5.11333±0.67343cd	3.96667±0.41435c	9.08±0.85719d
	WYZ	0.14147±0.01728a	0.01487±0.00239a	0.15633±0.01863a	26.05333±2.2178a	10.04667±0.55932b	36.1±2.17573a
	KYHZ	0.0059±0.000737111c	0.0036±0.000686375b	0.0095±0.00117c	3.38±0.23702d	7.13±0.51641bc	10.51±0.52035cd
	WYHZ	0.0276±0.00646c	0.0099±0.00218ab	0.0375±0.00837c	8.44±1.51879c	17.01±5.93971a	25.45±5.85524b
YH	KYH	0.06057±0.00747b	0.0247±0.00509b	0.08527±0.01204b	27.36±1.32872b	10.54667±1.12973a	37.90667±2.29317b

	WYH	0.29587±0.04369a	0.1211±0.02348a	0.41697±0.06563a	37.91667±2.15625a	12.20667±1.31535a	50.12333±3.34427a
YZ	KYZ	0.02717±0.00512b	0.00237±0.000319782b	0.02953±0.00533b	13.74333±1.72667b	3.63667±0.25086b	17.38±1.70319b
	WYZ	0.11247±0.01091a	0.0084±0.0017a	0.12087±0.01206a	28.87333±1.62748a	7.38333±0.60348a	36.25667±1.59886a
HZ	KHZ	0.05557±0.00809b	0.02508±0.00621b	0.08065±0.01352b	18.66±1.76414b	10.85167±0.87234b	29.51167±2.37812b
	WHZ	0.30563±0.06638a	0.2215±0.05567a	0.52713±0.11044a	27.67333±4.06549a	15.99±1.32264a	43.66333±5.21085a
YHZ	KYHZ	0.06103±0.01095b	0.02527±0.00672b	0.0863±0.0164b	20.45333±2.57153a	9.70333±1.32812a	30.15667±3.37225b
	WYHZ	0.29763±0.07421a	0.17427±0.05912a	0.4719±0.12885a	27.25±3.11436a	13.92±2.49312a	41.17±4.27951a

Y, H and Z represent *Vicia sepium* L. *Lolium perenne* L., and *Medicago sativa* L., respectively. a, b, c, d, and e indicate significant differences (P<0.05).

Table S2 The information of keystones in PT and PUT fungal network

Fungal Network	OTU	Network roles	Genus
PT	OTU3079	connectors	g__Pisolithus
	OTU2797	connectors	g__Arnium
	OTU413	module hubs	g__Trichophaeopsis
	OTU2502	module hubs	g__Didymella
	OTU1620	Network hubs	g__Strelitziana
PUT	OTU3391	connectors	g__Scopulariopsis
	OTU4520	connectors	g__Talaromyces
	OTU3567	connectors	g__Fusarium
	OTU754	connectors	g__Lentithecium
	OTU5310	connectors	g__Cutaneotrichosporon
	OTU4099	connectors	g__Myrmecridium
	OTU4312	module hubs	g__Alternaria
	OTU3567	module hubs	g__Fusarium
	OTU688	module hubs	g__Cerrena
	OTU5242	Network hubs	g__Fusarium

1.2 Supplementary Figures

Fig. S1 The difference in fungal community composition were analyzed using Multiple group comparisons based on Welch's t-test. *, ** and *** indicate $P<0.05$, $P<0.01$, $P<0.001$, respectively.

Fig. S2 Taxa at the genus level were displayed on the stacked column, and the taxa with relative abundances less than 1% were combined into others. (a) plant treatments. (b) plant combined with urban sludge treatments.

Fig. S3 The correlation and heatmap of modules in different networks. (a) and (b) represented the PT and PUT fungal network respectively. (c) and (d) represented the relationship between module and soil properties.

Fig. S4 Key species of all modules were shown based on Module Eigen-Gene analysis. (a) and (b) represent modules in PT and PUT fungal network respectively. The relative abundance of each species was showed in the heatmap of each sample.

Fig. S5 The relative abundance of keystone species in PT and PUT fungal network. (a) and (b) represented the PT and PUT fungal network respectively.
