# Supplementary figures and images for: Fungal community remediate quartz tailings soil under plant combined with urban sludge treatments
Source: Front Microbiol. 2023 Apr 20;14:1160960. doi: 10.3389/fmicb.2023.1160960 (PMC10157048; doi:10.3389/fmicb.2023.1160960)

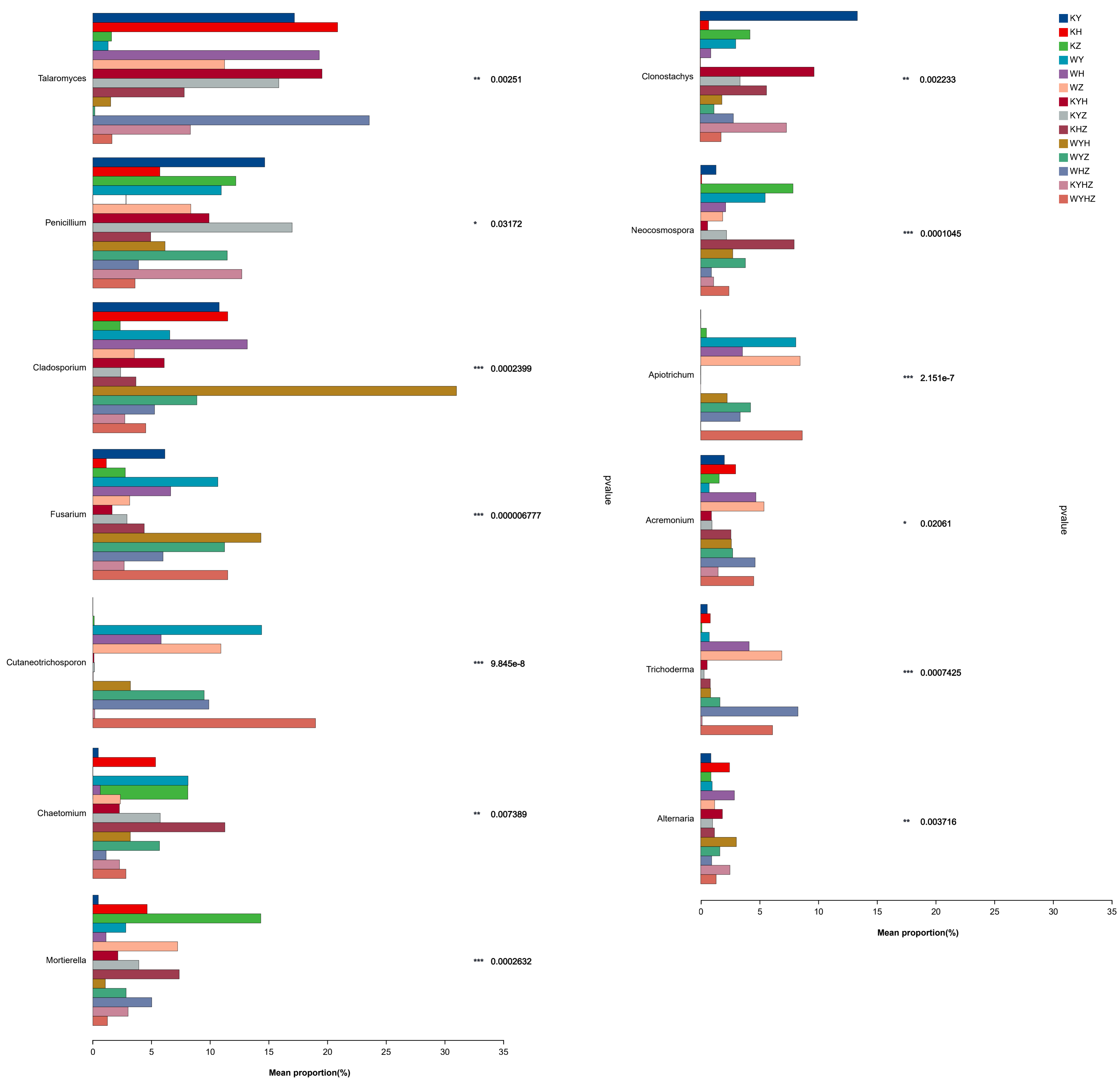

Supplement: Supplementary file 2 [file Data_Sheet_2.PDF]

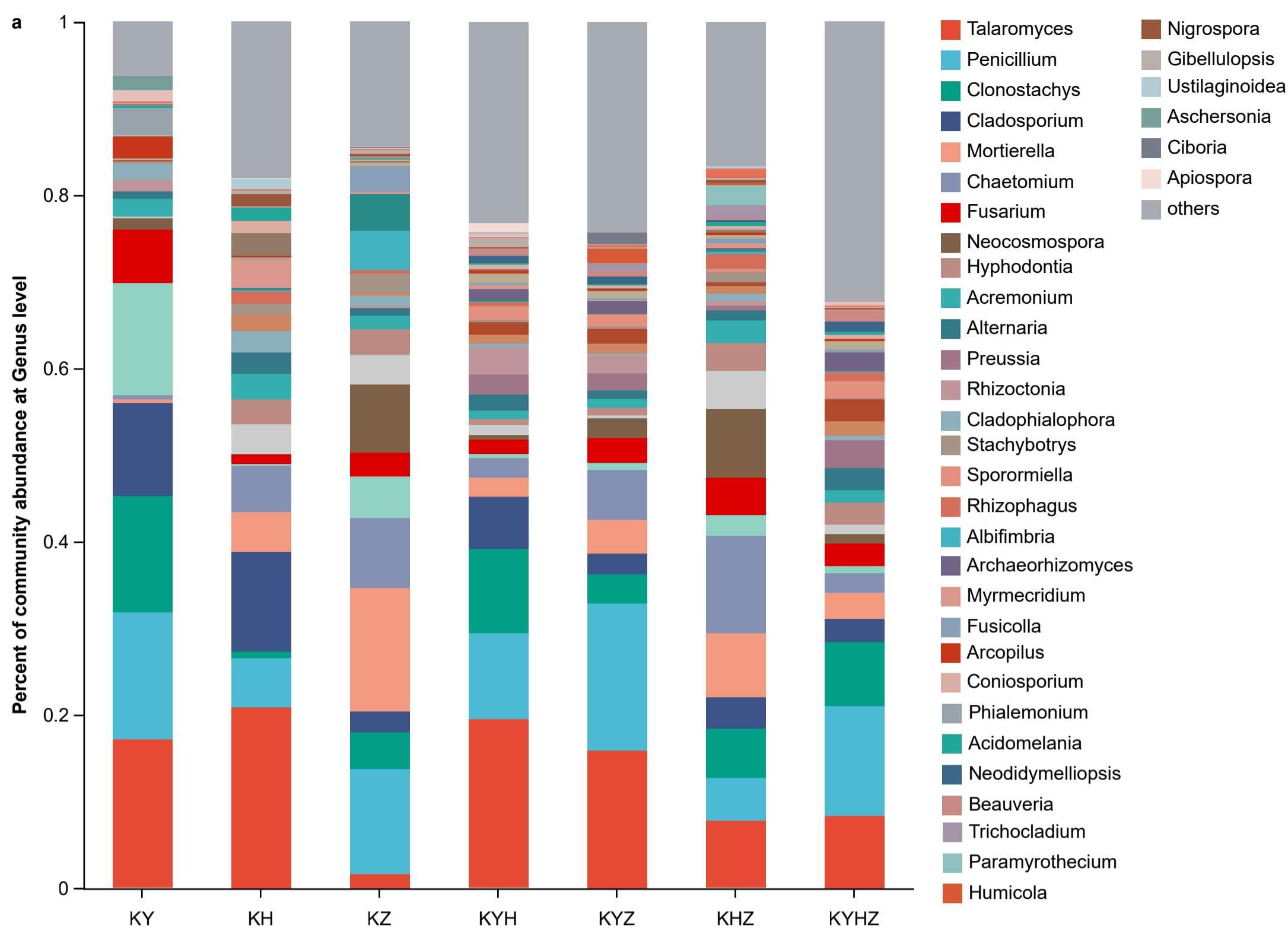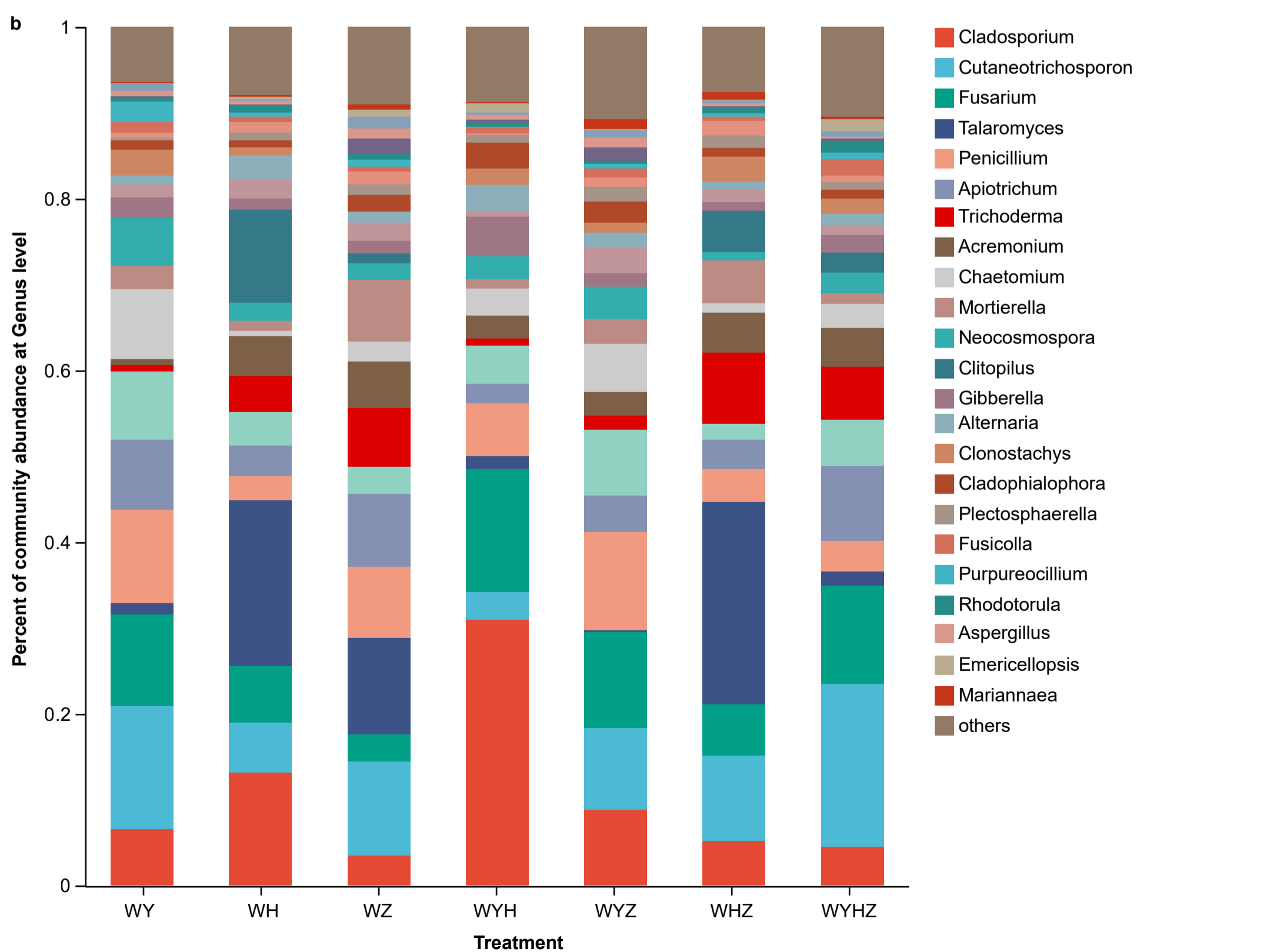

Supplement: Supplementary file 3 [file Data_Sheet_3.PDF]

a

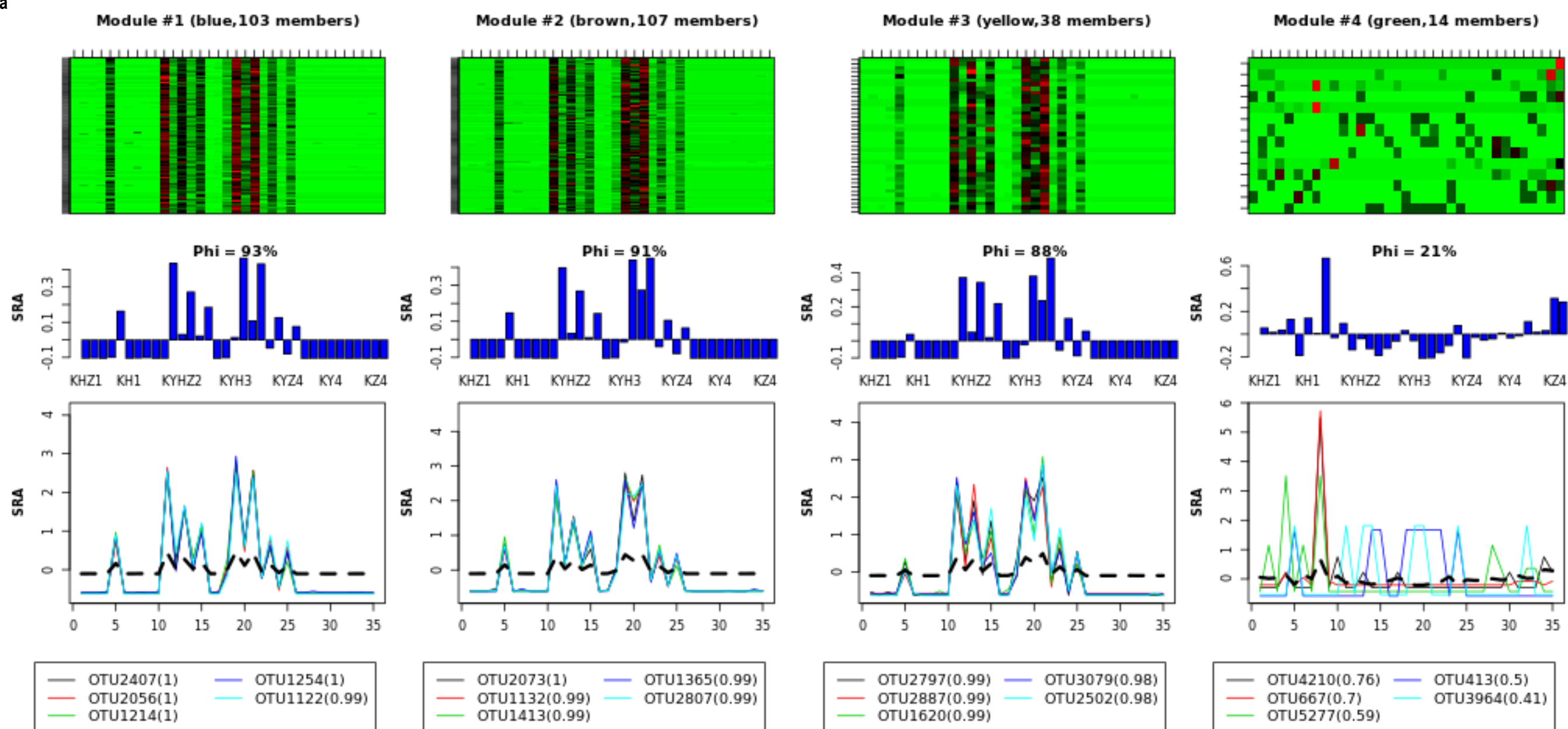

b

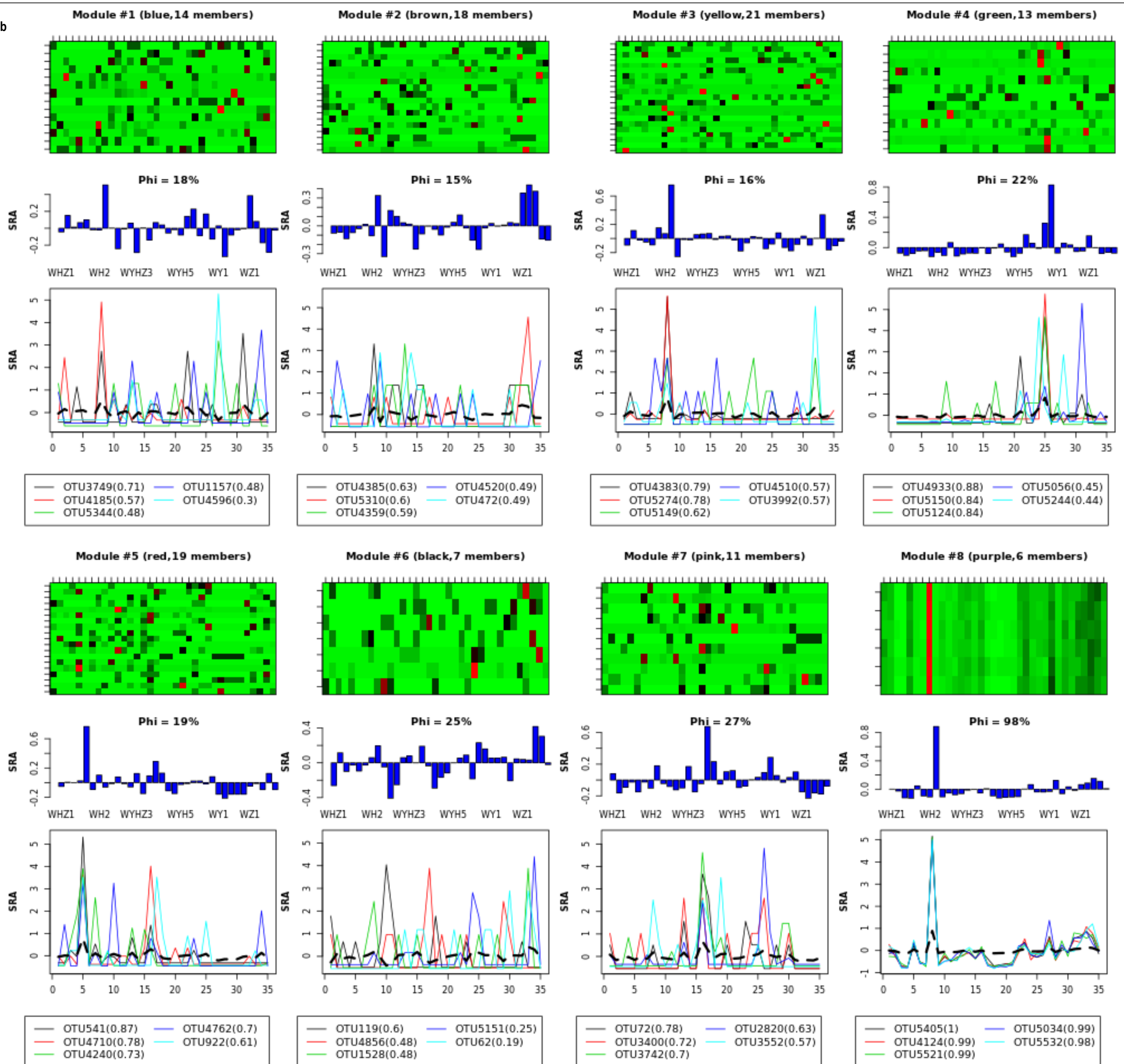

Supplement: Supplementary file 5 [file Data_Sheet_5.PDF]

**a**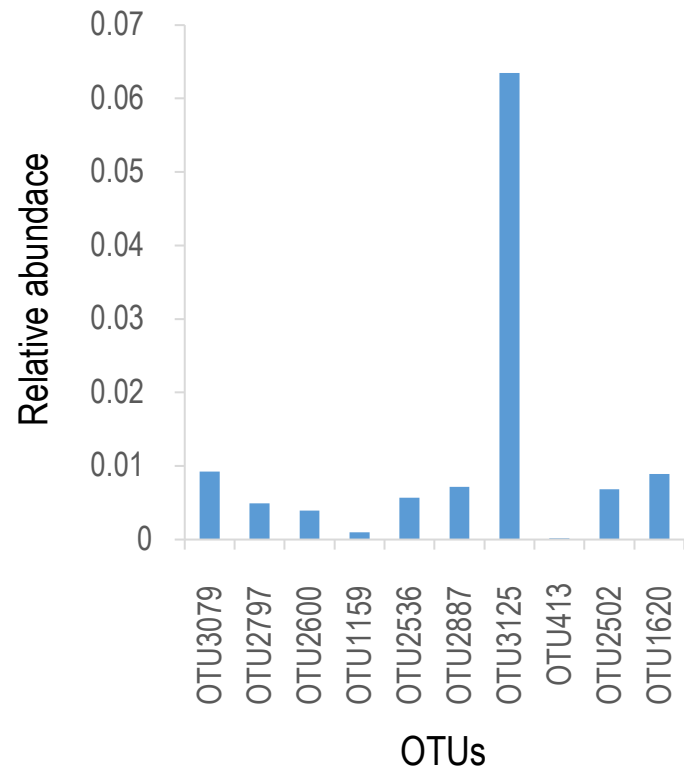**b**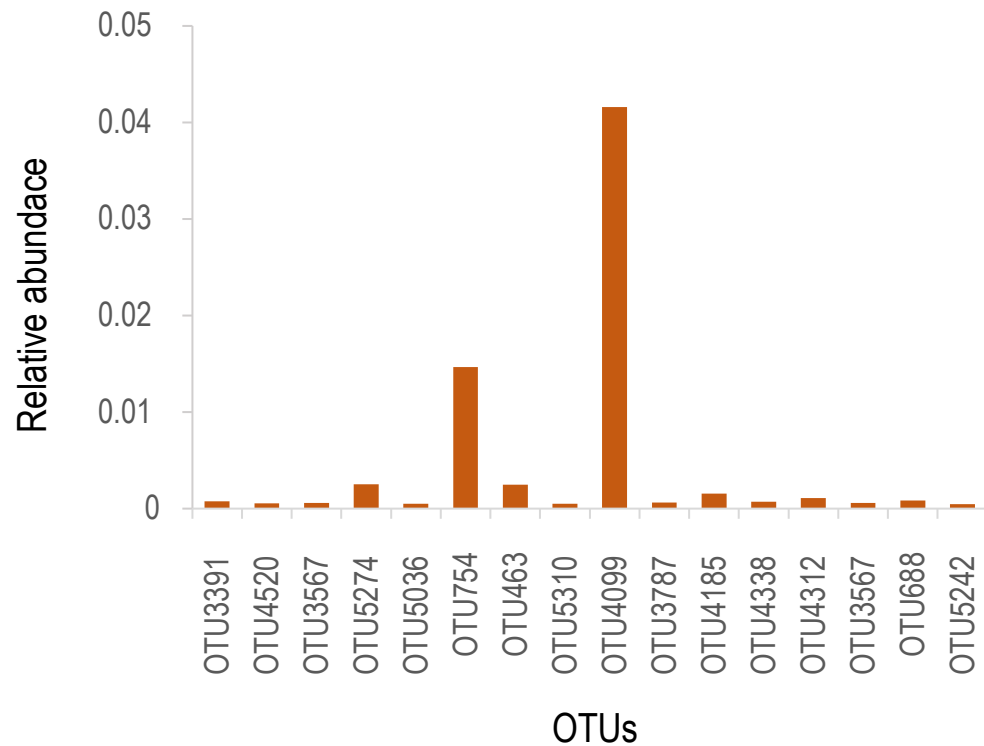

Supplement: Supplementary file 6 [file Data_Sheet_6.PDF]
