# Supplementary material for: Fungal community remediate quartz tailings soil under plant combined with urban sludge treatments
Source: Front Microbiol. 2023 Apr 20;14:1160960. doi: 10.3389/fmicb.2023.1160960 (PMC10157048; doi:10.3389/fmicb.2023.1160960)
Supplement: Supplementary file 4 [file Data_Sheet_4.pdf]

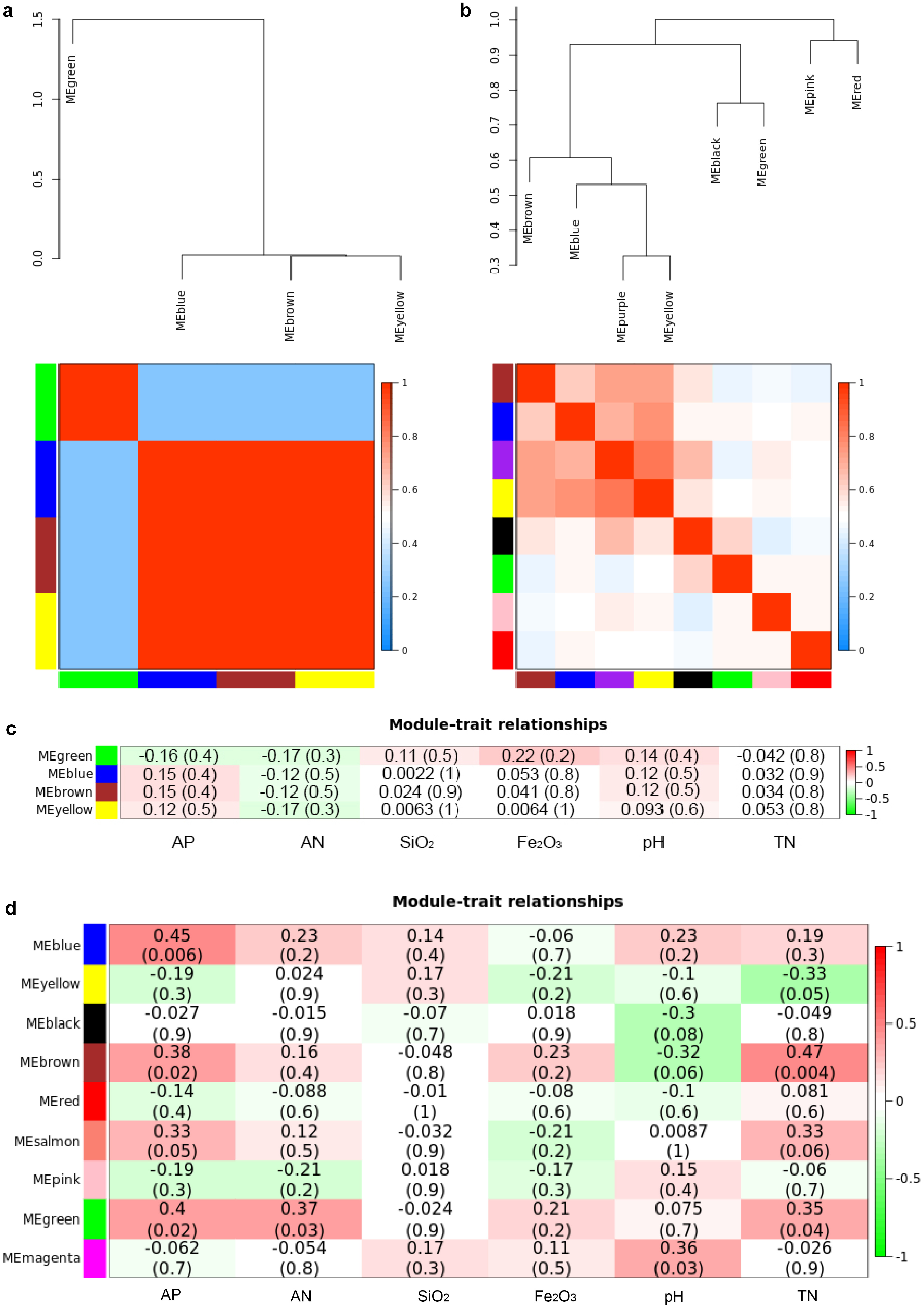
a
1.5
1.0
0.5
0.0
MEgreen
MEblue
MEbrown
MEyellow
1
0.8
0.6
0.4
0.2
0
b
1.0
0.9
0.8
0.7
0.6
0.5
0.4
0.3
MEbrown
MEblue
MEpurple
MEyellow
MEblack
MEgreen
MEpink
MEred
1
0.8
0.6
0.4
0.2
0
c
Module-trait relationships
MEgreen -0.16 (0.4) -0.17 (0.3) 0.11 (0.5) 0.22 (0.2) 0.14 (0.4) -0.042 (0.8)
MEblue 0.15 (0.4) -0.12 (0.5) 0.0022 (1) 0.053 (0.8) 0.12 (0.5) 0.032 (0.9)
MEbrown 0.15 (0.4) -0.12 (0.5) 0.024 (0.9) 0.041 (0.8) 0.12 (0.5) 0.034 (0.8)
MEyellow 0.12 (0.5) -0.17 (0.3) 0.0063 (1) 0.0064 (1) 0.093 (0.6) 0.053 (0.8)
AP AN SiO2 Fe2O3 pH TN
1
0.5
0
-0.5
-1
d
Module-trait relationships
MEblue 0.45 (0.006) 0.23 (0.2) 0.14 (0.4) -0.06 (0.7) 0.23 (0.2) 0.19 (0.3)
MEyellow -0.19 (0.3) 0.024 (0.9) 0.17 (0.3) -0.21 (0.2) -0.1 (0.6) -0.33 (0.05)
MEblack -0.027 (0.9) -0.015 (0.9) -0.07 (0.7) 0.018 (0.9) -0.3 (0.08) -0.049 (0.8)
MEbrown 0.38 (0.02) 0.16 (0.4) -0.048 (0.8) 0.23 (0.2) -0.32 (0.06) 0.47 (0.004)
MEred -0.14 (0.4) -0.088 (0.6) -0.01 (1) -0.08 (0.6) -0.1 (0.6) 0.081 (0.6)
MEsalmon 0.33 (0.05) 0.12 (0.5) -0.032 (0.9) -0.21 (0.2) 0.0087 (1) 0.33 (0.06)
MEpink -0.19 (0.3) -0.21 (0.2) 0.018 (0.9) -0.17 (0.3) 0.15 (0.4) -0.06 (0.7)
MEgreen 0.4 (0.02) 0.37 (0.03) -0.024 (0.9) 0.21 (0.2) 0.075 (0.7) 0.35 (0.04)
MEmagenta -0.062 (0.7) -0.054 (0.8) 0.17 (0.3) 0.11 (0.5) 0.36 (0.03) -0.026 (0.9)
AP AN SiO2 Fe2O3 pH TN
1
0.5
0
-0.5
-1
